# The role of hypertension in bone mineral density among males older than 50 years and postmenopausal females: evidence from the US National Health and Nutrition Examination Survey, 2005–2010

**DOI:** 10.3389/fpubh.2023.1142155

**Published:** 2023-06-15

**Authors:** Shuna Li, Li Li, Aozi Feng, Tao Huang, Chong Chen, Ningxia He, Liying Huang, Jun Lyu

**Affiliations:** ^1^Department of Clinical Research, The First Affiliated Hospital of Jinan University, Guangzhou, Guangdong, China; ^2^School of Public Health, Shaanxi University of Chinese Medicine, Xianyang, China; ^3^Guangdong Provincial Key Laboratory of Traditional Chinese Medicine Informatization, Guangzhou, Guangdong, China

**Keywords:** bone mineral density, hypertension, low bone mass, osteoporosis, gender, NHANES

## Abstract

**Background:**

Hypertension is a significant chronic disease that has been linked with bone mineral density (BMD) in various studies. However, the conclusions are contradictory. The purpose of our study was to identify the bone mineral density (BMD) of postmenopausal females and males older than 50 years with hypertension.

**Methods:**

This cross-sectional study of 4,306 participants from the 2005–2010 US National Health and Nutrition Examination Survey explored the relationship between BMD and hypertension. Participants who had a mean systolic blood pressure (SBP) ≥140 mmHg, or a mean diastolic blood pressure (DBP) ≥90 mmHg, or were taking any prescribed medicine for high blood pressure were defined as having hypertension. BMD values were measured at the femoral neck and lumbar vertebrae as the primary outcome. Weight general linear model was used to describe the status of BMD in patients with hypertension. Weighted multivariate regression analysis was conducted to demonstrate the association between hypertension and BMD. Weighted restricted cubic spline (RCS) was used to assess the relationship between BMD and SBP and DBP.

**Results:**

Our study found that there was a positive association between hypertension and lumbar BMD and the lumbar BMD was significantly higher in the presence of hypertension than in the control group in both males (1.072 vs. 1.047 g/cm^2^) and females (0.967 vs. 0.938 g/cm^2^; both *p* < 0.05), but a similar pattern was not found in the femoral neck. Meanwhile, lumbar BMD was positively associated with SBP and negatively associated with DBP both in males and females. The prevalence of low bone mass and osteoporosis at the lumbar vertebrae was lower in male patients with hypertension than in the control group. However, no difference was observed among postmenopausal females between the hypertension and control groups.

**Conclusions:**

Hypertension was associated with higher BMD at the lumbar vertebrae in both males older than 50 years and postmenopausal females.

## Introduction

Hypertension is a common public health problem whose prevalence increases with age (1). Osteoporosis is a bone disease characterized by a decrease in bone mass and microstructure, and bone tissue (2, 3). Several studies have indicated that age plays an important role in osteoporosis, and osteoporosis increases fracture occurrence, which poses a considerable disease burden for society. In 2005, the estimated direct medical costs associated with treating osteoporosis in the United States ranged from $13.7 billion to $20.3 billion (3). The number of annual cases of osteoporotic fracture is estimated to be exceed three million with a cost of $25.3 billion by 2025 (3–5).

Males older than 50 years and postmenopausal females are a population with a high risk of hypertension and osteoporosis. Bone mineral density (BMD) is an important clinical parameter of human bone strength and predicting bone fracture risk, and is the current gold standard for diagnosing osteoporosis (6). Some researchers have found an association between fractures and BMD values in the lumbar vertebrae and femoral neck (7). A meta-analysis of prospective studies found that BMD measurements at the spine have an acceptable predictive ability for spine fractures (RR 2.3, 1.9–2.8), and hip BMD can predict hip fractures (RR 2.6, 2.0–3.5) with one SD drop in bone density (8). However, a wide overlap in BMD between the patients with and without incident fracture exists. More attention should therefore be given to the BMD status of males ≥50 years old and postmenopausal females with hypertension.

However, previous studies have produced conflicting conclusions on the association between blood pressure and BMD. Tsuda et al. (9) and Hong et al. (10) considered hypertension to be inversely associated with BMD. Other studies found no association between blood pressure and BMD in postmenopausal females (11–13). The population-based Canadian Multicentre Osteoporosis Study, which was a cross-sectional cohort study of males and females older than 50 years (5,566 females and 2,187 males), found that hypertension was associated with higher BMD (14). It has similarly been reported that a positive association was found between lumbar BMD and systolic blood pressure (SBP) in 2,000 females and 2,000 males (15).

Given the different results of these studies and considering different countries and sample sizes may affect the results of BMD status in hypertension, and lumbar BMD and femoral neck are the most common sites of osteoporosis. Measuring the BMD of the femoral neck and lumbar spine can evaluate changes in bone metabolism and the degree of osteoporosis, which have special importance and significance. Therefore, we used the 2005–2010 National Health and Nutrition Examination Survey (NHANES) cross-sectional study, a nationally representative sample of the US, to explore the BMD status of lumbar and femoral neck among postmenopausal females and males older than 50 years with and without hypertension in the United States. This study may provide new ideas for the control of hypertension in the middle-aged and elderly.

## Method

### Study design and study population

NHANES is conducted by the National Center for Health Statistics (NCHS) of the Centers for Disease Control and Prevention and is designed to evaluate the health and nutritional status of adults and children in the United States. NHANES is a cross-sectional study, and its publicly available data cover interviews and medical examinations, including demographic, dietary, examination, laboratory, and questionnaire data. Some researchers have provided detailed descriptions of relevant public databases (16).

**Figure 1:**
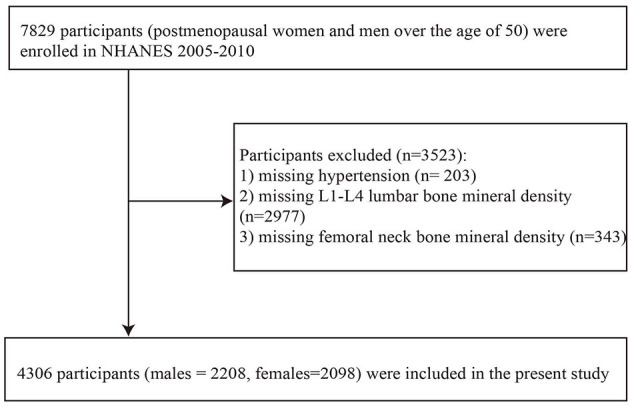
Flowchart inclusion of study participants. BMI, body mass index; family PIR, family poverty income ratio.

The data of the present study was obtained from the consecutive versions of the NHANES, and the reason for choosing these three consecutive versions is that the BMD values of the proximal femur and lumbar vertebrae have been measured using a fan-beam DXA up until now. The study focused on postmenopausal females and males older than 50 years. The initial study sample enrolled 7,829 individuals. From this initial sample, we excluded those with missing data on hypertension, L1–L4 vertebrae BMD, femoral neck BMD, total calcium, body mass index (BMI), physical activity status, recent smoking status, marriage, family poverty income ratio (PIR), education, or self-reported cardiovascular disease (congestive heart failure, coronary heart disease, angina pectoris, heart attack, and stroke) or other diseases (arthritis, emphysema, and chronic bronchitis). The remaining 4,306 participants (2,208 males and 2,098 females) were analyzed in the present study. The detailed data screening process is shown in Figure 1.

### BMD measurement

Hologic QDR 4500A fan-beam densitometers (Hologic, Bedford, MA) were used to measure the femoral neck and anterior-posterior lumbar vertebrae according to the recommended procedures. Within the 2005–2010 NHANES, the femur scans were estimated using version 12.4 of Hologic Discovery, and the lumbar scans were calculated using APEX (version 3.0) software. The mean value among the first to the fourth lumbar vertebrae was taken as the value of lumbar BMD. T-scores were also used to describe the osteoporosis statuses of males older than 50 years and postmenopausal females, calculated as (measured BMD – mean BMD of reference group)/(SD of BMD in the reference group) (17). Based on WHO recommendations (18), we selected 20–29 non-Hispanic white females from NHANES III as a reference group for femoral neck BMD (19). For the lumbar BMD reference group, we selected 30-year-old white females from the DXA manufacturer reference database (20). T-scores were divided into three categories: ≥-1.0, < -1.0 and >-2.5, and ≤ -2.5, which were designated as normal skeletal status, low bone mass, and osteoporosis, respectively (21).

The details of blood pressure measurements have been described in NHANES (https://wwwn.cdc.gov/Nchs/Nhanes/2005-2006/BPX_D.htm). The data of the first, second, and third blood pressure measurements were used for the present analyses. If the blood pressure of a participant was measured three times, the mean SBP and mean diastolic blood pressure (DBP) were defined as the mean of the three readings; if their blood pressure was measured twice, the mean SBP and mean DBP were the mean of the two readings; if they were measured once, then it was the mean SBP and the mean DBP.

### Hypertension definition

Participants who had a mean SBP ≥140 mmHg, or a mean DBP ≥90 mmHg, or took any prescribed medicine for high blood pressure were defined as having hypertension, which was based on the Joint National Committee [JNC]7 guideline (22). In NHANES, a participant was considered to have high blood pressure if they answered “yes” to the question “Are you now taking prescribed medicine for high blood pressure?”.

### Covariates

Additional information included baseline data such as demographic variables (e.g., age, sex, race, marital status, family PIR, education, and disease history), lifestyle variables (e.g., recent smoking status, BMI, and physical activity status), and biochemical indicators (serum calcium).

Among demographic variables, marital status was classified as living with others (married or living with a partner) and living alone (widowed, divorced, separated, or never married). Family PIR was grouped into three categories: low (≤ 1), middle (>1 and < 4), and high (≥4) (23). Education was divided into lower than high school, high school or equivalent, and college or above. Some self-reported diseases were included, such as arthritis, congestive heart disease, coronary heart disease, angina pectoris, heart attack, and stroke.

For recent smoking status, we analyzed the smoking history of participants during the past 5 days. Smoking was defined as any product containing nicotine, such as cigarettes, pipes, cigars, chewing tobacco, snuff, nicotine patches, or nicotine gum. Body measurement data (e.g., BMI) were collected by trained health technicians. In addition, physical activity status was demonstrated by self-reported moderate/vigorous recreational physical activity (RPA). In the 2005–2006 NHANES data, self-reported moderate/vigorous RPA was ascertained if the participant answered “yes” to the following questions: “Over the past 30 days, did [you/study participant (SP)] do any vigorous activities for at least 10 min that caused heavy sweating, or large increases in breathing or heart rate? Some examples are running, lap swimming, aerobics classes or fast bicycling,” and “Over the past 30 days, did (you/SP) do moderate activities for at least 10 min that caused only light sweating or a slight to moderate increase in breathing or heart rate? Some examples are brisk walking, bicycling for pleasure, golf, and dancing.” In the 2007–2010 NHANES data, the relevant questions were “(Do you/Does SP) do any vigorous-intensity sports, fitness, or recreational activities that cause large increases in breathing or heart rate like running or basketball for at least 10 min continuously?” and “(Do you/Does SP) do any moderate-intensity sports, fitness, or recreational activities that cause a small increase in breathing or heart rate such as brisk walking, bicycling, swimming, or golf for at least 10 min continuously?”.

### Statistical analyses

Variables conforming to a normal distribution were expressed as mean ± standard error (SE), and categorical variables were expressed as numbers and weighted percentages. The weighted *t*-test or chi-square test was used to analyze baseline information in the control and hypertension groups. Considering that BMD varies greatly between sexes, we divided the participants into male and female subgroups to analyze the differences in BMD distribution. Weighted multivariate linear regression was used to demonstrate the association between hypertension and BMD of the femoral neck and lumbar vertebrae. Model 1 did not adjust any variables, model 2 adjusted for age, race, and BMI, and model 3 adjusted for age, race, marriage, BMI, family PIR, education, insurance, recent smoking status, physical activity status, total calcium, arthritis, congestive heart failure, coronary heart disease, angina/angina pectoris, heart attack, and stroke. Weight general linear model was performed to describe the mean and SE values of the BMDs of femoral neck and lumbar vertebrae in the hypertension and control groups. The prevalence rates of normal skeletal status, low bone mass, and osteoporosis in at-risk populations (males older than 50 years and postmenopausal females) were analyzed according to types of hypertension. In addition, the relationship between BMD of femoral neck and lumbar vertebrae and SBP and DBP was assessed by weighted restricted cubic spline (RCS).

Considering the possible impact of antihypertensive drugs, glucocorticoids (prednisone or cortisone), thyroid function on BMD, sensitivity analysis was conducted to further verify the robustness of the results by excluding the population taking hypertensive drugs, people taking prednisone or cortisone, and people with thyroid problems.

All statistics were completed in the “survey” package of the R software. Probability values of *p* < 0.05 were considered to be statistically significant.

## Results

### The baseline characteristics of subjects

The final analysis included 4,306 participants. Baseline characteristics of the eligible participants are listed in Table 1. There were 2,341 participants in the hypertension group and 1,965 in the control group.

**Table 1:** Baseline characteristics of postmenopausal females and male participants over 50 years of age from the US National Health and Nutrition Examination Survey (US NHANES).

**Characteristics**	**Control (*n* = 1,965)**	**Hypertension (*n* = 2,341)**	***p-*value**
Age (years)	57.33 ± 0.30	63.44 ± 0.29	< 0.001
**Gender**			0.687
Male	1,036 (48.12%)	1,172 (47.32%)	
Female	929 (51.87%)	1,169 (52.68%)	
**Ethnicity or race**			< 0.001
Mexican American	368 (5.57%)	353 (4.83%)	
Other Hispanic	180 (3.25%)	179 (2.79%)	
Non-Hispanic White	1,052 (78.96%)	1,183 (75.76%)	
Non-Hispanic Black	293 (7.01%)	549 (11.84%)	
Other race	72 (5.21%)	77 (4.78%)	
**Marital status**			0.350
Live alone	1,279 (69.44%)	1,444 (67.77%)	
Live with others	686 (30.56%)	897 (32.23%)	
**Family PIR**			< 0.001
High	694 (49.99%)	611 (37.86%)	
Medium	954 (41.08%)	1,377 (53.96%)	
Low	317 (8.93%)	353 (8.18%)	
**Education**			< 0.001
College or above	965 (59.58%)	984 (51.60%)	
High school or equivalent	443 (24.87%)	574 (26.78%)	
Less than high school	557 (15.56%)	783 (21.62%)	
**Smoking**			< 0.001
Yes	481 (24.78%)	433 (17.44%)	
No	1,484 (75.22%)	1,908 (82.56%)	
BMI (kg/m^2^)	27.20 ± 0.16	29.03 ± 0.15	< 0.001
Vigorous/moderate RPA	957 (56.53%)	1,009 (50.46%)	0.001
Serum calcium (mmol/L)	2.367 ± 0.003	2.37 ± 0.004	0.015
Arthritis	649 (33.19%)	1,042 (43.18%)	< 0.001
Congestive heart disease	40 (1.64%)	152 (5.40%)	< 0.001
Coronary heart disease	82 (3.30%)	206 (8.62%)	< 0.001
Angina/angina pectoris	49 (2.00%)	138 (5.30%)	< 0.001
Heart attack	88 (3.89%)	217 (7.78%)	< 0.001
Stroke	47 (2.15%)	173 (6.19%)	< 0.001

Values with normal distribution were expressed as weighted mean and stand error (SE) and categorical variables were expressed as number and weighted proportion.

BMI, body mass index; PIR, poverty income ratio; RPA, recreational physical activity.

The age distribution differed significantly between the hypertension and control groups (*p* < 0.001): 63.44 ± 0.29 and 57.33 ± 0.30 years, respectively. There were also significant differences between the control group and hypertension group in race, BMI, education, family PIR, physical activity status, recent smoking status, serum calcium, cardiovascular disease history (congestive heart disease, coronary heart disease, angina pectoris, heart attack, and stroke), and arthritis history, but not marital status and gender.

### The mean BMD of hypertension and control group

The mean BMDs of different groups in the 2005–2010 NHANES are listed in Table 2. There were significantly higher lumbar spine BMD in hypertensive male compared to control group that remained significant without adjustment (*p* < 0.001) and after adjusting for age, race/ethnicity, and BMI (*p* = 0.003). The differences persisted further after adjusting for marriage, family PIR, education, recent smoking status, physical activity status, total calcium, arthritis, congestive heart failure, coronary heart disease, angina/angina pectoris, heart attack, and stroke (*p* = 0.001). Among males, the hypertension group had higher lumbar BMD than the control group (model 3: 1.072 vs. 1.047 g/cm^2^). A similar result was observed in the lumbar BMD of females after the adjustments of model 3 (hypertension vs. control: 0.967 vs. 0.938 g/cm^2^, *p* = 0.003). However, the difference in femoral neck BMD between the control and hypertension groups was not significant for both males and females (*p* > 0.05).

**Table 2:** Mean BMD (g/cm^2^) according to the type of hypertension in NHANES 2005 to 2010.

	**Control**	**Any hypertension**	***p-*value**
**Femur neck BMD (g/cm** ^2^ **)**			
**Male**			
Model 1	0.809 ± 0.003	0.821 ± 0.005	0.063
Model 2	0.813 ± 0.004	0.817 ± 0.005	0.547
Model 3	0.812 ± 0.004	0.817 ± 0.005	0.455
**Female**			
Model 1	0.738 ± 0.005	0.734 ± 0.005	0.458
Model 2	0.732 ± 0.005	0.741 ± 0.005	0.136
Model 3	0.731 ± 0.005	0.741 ± 0.005	0.094
**Lumbar spine BMD (g/cm** ^2^ **)**			
**Male**			
Model 1	1.038 ± 0.005	1.081 ± 0.005	< 0.001
Model 2	1.048 ± 0.005	1.071 ± 0.006	0.003
Model 3	1.047 ± 0.005	1.072 ± 0.005	0.001
**Female**			
Model 1	0.943 ± 0.007	0.961 ± 0.005	0.043
Model 2	0.939 ± 0.006	0.965 ± 0.006	0.005
Model 3	0.938 ± 0.006	0.967 ± 0.006	0.003

Values are the means ± standard error by weighted general linear models.

Model 1: adjusted without any variables.

Model 2: adjusted for age, race/ethnicity, and BMI.

Model 3: adjusted for age, race/ethnicity, marriage, BMI, family PIR, education, recent smoking status, physical activity, total calcium, arthritis, congestive heart failure, coronary heart disease, angina/angina pectoris, heart attack, and stroke.

### The association between BMD and hypertension

The associations between BMD and hypertension are demonstrated in Table 3. There is a positive association between hypertension and lumbar BMD in males. Compared with non-hypertension, the hypertension participants have a slightly higher BMD in males of the lumbar spine in three models (model 3: β = 0.025, 95%CI = 0.011, 0.039, *p* = 0.001). In females, we found a similar positive association between hypertension and BMD of the lumbar spine after the fully adjusted model (model 3: β = 0.028, 95%CI = 0.010, 0.046, *p* = 0.003). However, we did not find a significant association between hypertension and BMD of the femur neck both in males and females (*p* > 0.05).

**Table 3:** Weighted multivariate linear regression of association between hypertension and BMD in males and females.

	**Male**			**Female**		
	**β**	**95% CI**	***p*-value**	**β**	**95% CI**	***p*-value**
**Femur neck BMD g/cm** ^2^						
Model 1	0.011	0, 0.023	0.063	−0.005	−0.018, 0.009	0.458
Model 2	0.004	−0.010, 0.018	0.547	0.009	−0.003, 0.022	0.136
Model 3	0.005	−0.008, 0.018	0.455	0.011	−0.002, 0.023	0.094
**Lumbar spine BMD g/cm** ^2^						
Model 1	0.043	0.030, 0.056	< 0.001	0.018	0.001, 0.035	0.043
Model 2	0.023	0.008, 0.038	0.003	0.026	0.008, 0.043	0.005
Model 3	0.025	0.011, 0.039	0.001	0.028	0.010, 0.046	0.003

Model 1: adjusted without any variables.

Model 2: adjusted for age, race/ethnicity, and BMI.

Model 3: adjusted for age, race/ethnicity, marriage, BMI, family PIR, education, recent smoking status, physical activity, total calcium, arthritis, congestive heart failure, coronary heart disease, angina/angina pectoris, heart attack, and stroke.

### The association between BMD and SBP, DBP

**Figure 2:**
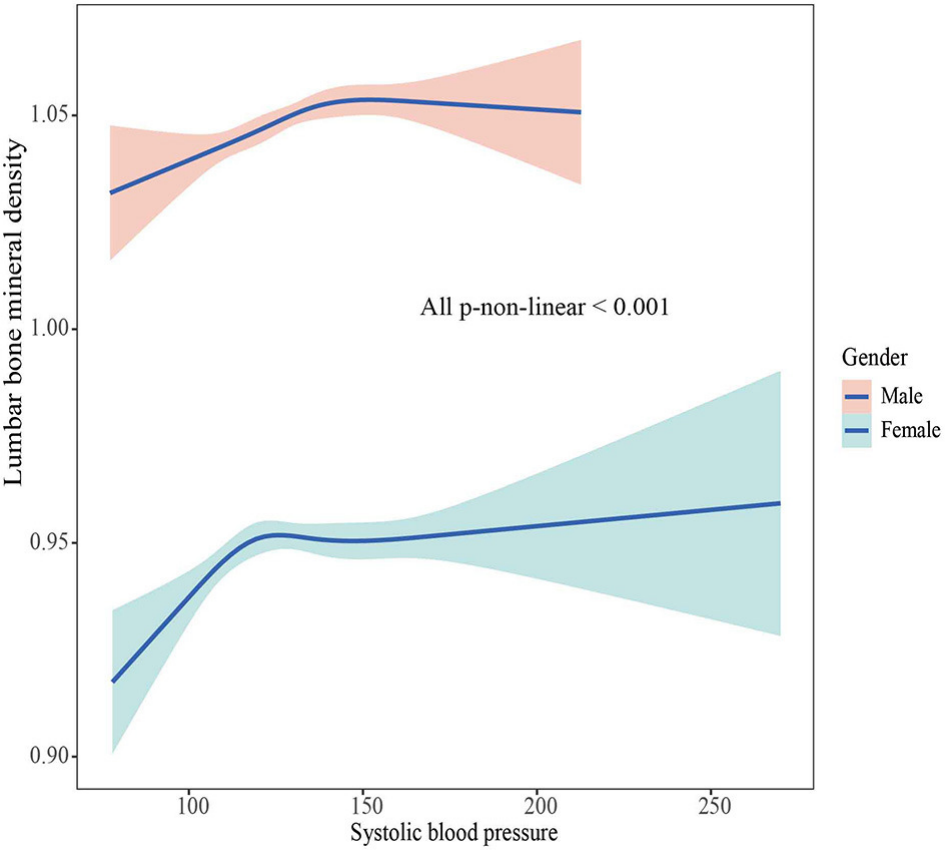
The association between lumbar bone mineral density and systolic blood pressure.

**Figure 3:**
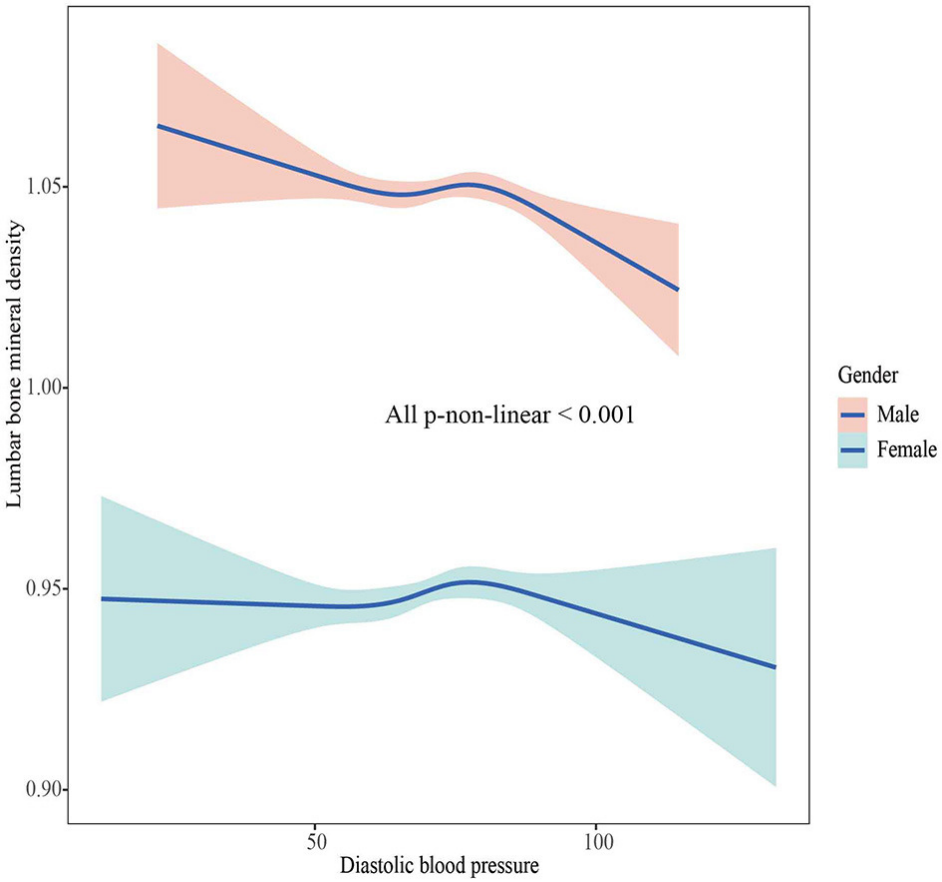
The association between lumbar bone mineral density and diastolic blood pressure.

**Figure 4:**
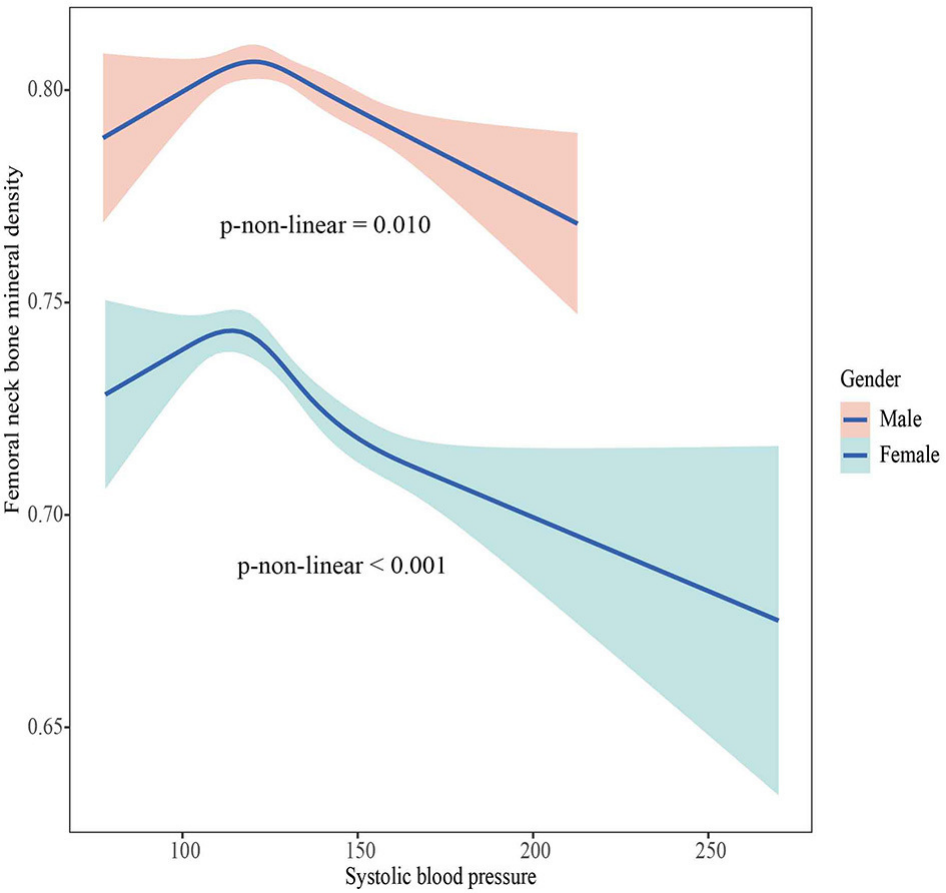
The association between femoral neck bone mineral density and systolic blood pressure.

**Figure 5:**
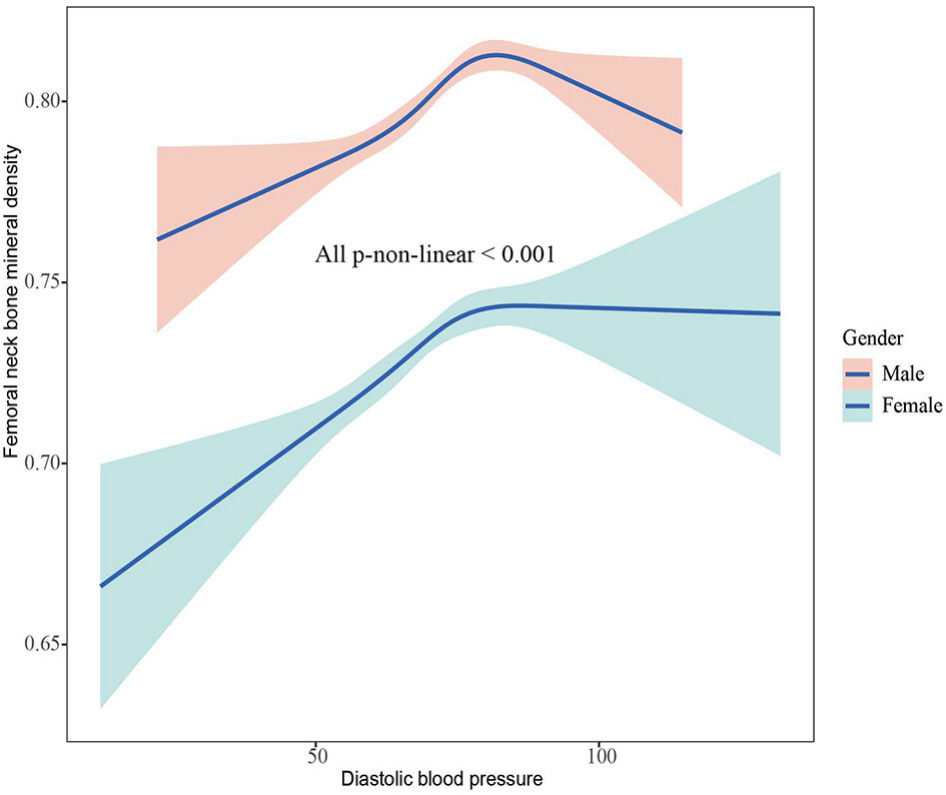
The association between femoral neck bone mineral density and diastolic blood pressure.

The associations between BMD and SBP, DBP are shown in Figures 2–5. A positive correlation was observed between lumbar BMD and SBP for male and female (*p*-non-linear < 0.001). Meanwhile, we found a negative correlation between lumbar BMD and DBP both in male and female (*p*-non-linear < 0.001). However, the correlation between femoral neck BMD and SBP and DBP in both male and female was in the opposite direction as the correlation between lumbar BMD and SBP and DBP (*p*-non-linear < 0.050).

### The prevalence of normal, low bone mass, or osteoporosis

Table 4 presents a detailed list of the distribution of normal skeletal status, low bone mass, and osteoporosis. The prevalence rates of lumbar low bone mass (hypertension vs. control: 16.52% vs. 20.18%) and osteoporosis (hypertension vs. control: 1.57% vs. 4.20%) among for males were significantly lower in the hypertension group than in the control group (*p* = 0.004). There was no significant difference between hypertension and control groups for males in low bone mass and osteoporosis of the femoral neck. In females, there was no significant difference between the control group and the hypertension group regarding femoral neck and lumbar BMDs.

**Table 4:** Prevalence of normal, low bone mass, or osteoporosis in the at-risk population (male participants aged ≥50 years and postmenopausal females) between control and hypertension.

	**Control *N* (%)**	**Any hypertension *N* (%)**	***p-*value**
**Femur neck** ***T*****-score**			
**Total**			0.294
Normal	1,150 (57.98%)	1,340 (57.08%)	
Low bone mass	753 (38.22%)	880 (37.97%)	
Osteoporosis	82(3.80%)	121 (4.94%)	
**Male**			0.943
Normal	704 (69.74%)	807 (69.65%)	
Low bone mass	314 (28.84%)	343 (28.76%)	
Osteoporosis	18 (1.42%)	22 (1.59%)	
**Female**			0.330
Normal	446 (47.06%)	533 (45.80%)	
Low bone mass	419 (46.92%)	537 (46.25%)	
Osteoporosis	64 (6.01%)	99 (7.96%)	
**Lumbar spine** ***T*****-score**			
**Total**			0.013
Normal	1,199 (62.11%)	1,546 (67.90%)	
Low bone mass	612 (30.68%)	613 (25.99%)	
Osteoporosis	154 (7.21%)	182 (6.11%)	
**Male**			0.004
Normal	762 (75.62%)	945 (81.92%)	
Low bone mass	233 (20.18%)	203 (16.52%)	
Osteoporosis	41 (4.20%)	24 (1.57%)	
**Female**			0.102
Normal	437 (49.58%)	601 (55.32%)	
Low bone mass	379 (40.42%)	410 (34.50%)	
Osteoporosis	113 (10.00%)	158 (10.18%)	

### Sensitivity analysis

After excluding the participants with taking antihypertensive drugs, people taking prednisone or cortisone, and people with thyroid problems, sensitivity analysis did not find substantial changes (Supplementary Tables 1–6).

## Discussion

This was a descriptive study aiming to demonstrate the BMD status in hypertension among postmenopausal females and males older than 50 years. We found that lumbar BMD was higher in the hypertension group than in the control group, and there was a positive association between lumbar BMD and hypertension in males and females, but a similar pattern was not found for femoral neck BMD. In addition, lumbar BMD was positively associated with SBP and negatively correlated with DBP among males and females. Moreover, low bone mass and osteoporosis at the lumbar vertebrae were less prevalent in males with hypertension than in the control group. However, that difference was not observed among postmenopausal females.

It was difficult to explain why the BMD of the hypertension group was higher than that in the group without hypertension at the lumbar vertebrae in both males and females. The biological mechanisms underlying the high BMD in hypertensive patients are currently unclear, and one possible biological mechanism is the increased mechanical loading of the skeleton. Patients with hypertension tend to have higher body weight and increased muscle mass and have more developed skeletal muscle forces as the lumbar spine bears the weight of the body, which results in increased mechanical forces exerted on the bones. These forces may stimulate bone growth and mineralization, leading to increased BMD (24). Another biological mechanism that may contribute to high bone density in patients with hypertension is hormonal changes. Hypertension is associated with increased levels of various hormones, such as parathyroid hormone (25), which plays a key role in bone remodeling. Increased levels of parathyroid hormone stimulate bone formation, which leads to an increase in BMD (26, 27). Meanwhile, the hypertension group had a lower total proportion of low bone mass and osteoporosis than the control group, although this difference was not significant among females. In addition, the sample population had adequate power (in terms of the sample size and representativeness), meaning that the results were unlikely to have been caused by chance. More importantly, our results were supported by those of studies with similar research designs. Hanley et al. (14) conducted a cross-sectional study (the population-based Canadian Multicentre Osteoporosis Study) that included 5,566 females and 2,187 males 50 years of age or older, suggesting that hypertension was associated with higher BMD, and compared with those without hypertension, there were notable differences at the lumbar vertebrae in males (+0.028 g/cm^2^) and in females (+0.022 g/cm^2^). Woo et al. (15) found that lumbar BMD had a positive association with SBP in another cross-sectional study that involved analyzing 2,000 males and 2,000 females aged 65 years performed by the School of Public Health and Primary Care of the Chinese University of Hong Kong. A recent systematic review and meta-analysis found that essential hypertension cannot reduce the lumbar BMD in non-Asian populations (28). In addition, a longitudinal study that included 1,032 males and 1,701 females aged 50 years or older found that males with hypertension were associated with higher femoral neck BMD than those without hypertension (0.94 vs. 0.92 g/cm^2^, *p* = 0.02). That study supported our conclusion in that BMD in males with high blood pressure may be higher than in those without (29). However, our results conflict with those of several studies, such as an inverse association between hypertension and BMD in males (30–32).

The postmenopausal females showed more inconsistent results. Many researchers have found no difference between participants with hypertension and healthy participants based femoral neck or lumbar vertebrae BMD (11, 12, 33, 34). Lee et al. (34) found no correlation between hypertension and low BMD at either the lumbar vertebrae (L1–L4) or the femoral neck among elderly African American females (age ≥ 65 years) after adjusting for certain confounding factors. Furthermore, an inverse association was found between hypertension and BMD in different studies (35–37). There were some differences between these conclusions and those in our study.

Given that previous results differed to ours, we consider the following possibilities: firstly, in our study, participants with hypertension may have been more likely to pay attention to a healthy diet or deliberately look for a healthier lifestyle (38). Protein intake was a positive predictor of lumbar BMD after controlling for dietary sulfate content (39). In females, lycopene intake had a protective effect against a 4-year loss of lumbar BMD (40). Besides, lumbar vertebrae BMD of postmenopausal females was positively associated with Mediterranean diet score, which was assessed based on the intake of cereals, vegetables, fruits, meats, dairy products, fish, red wine, and olive oil (41). Secondly, the circadian system and sleeping may play important roles in bone resorption and formation (42). Fu et al. (43) found that shorter sleep time was closely related to low BMD, especially in females older than 45 years. Compared with those who slept for 7 h per night (reference), females who slept for < 5 h per night had lower BMD (mean decrease from 0.012 to 0.018 g/cm^2^) (44). The females who slept 5 h or less per night had a higher risk of low bone mass and osteoporosis at the lumbar vertebrae (odds ratio = 1.28, 95% confidence interval = 1.02–1.60). Thirdly, the age-related variations in BMD measurements may cause some differences. Notably, for middle-aged and elderly people, lower blood pressure does not mean better body health. Keeping blood pressure at a level that maintains body health may be the best choice. Finally, the size and representativeness of the sample may also cause bias in the results.

Nutrition, sleeping, BMD measurement methods, and sample size were external factors that explained the present study results, but the specific internal mechanism is not yet clear. It is worth noting that some researchers mentioned the role of ghrelin. Ghrelin not only regulated blood pressure but also had a beneficial effect on bone mass (45, 46). In postmenopausal females with hypertension, ghrelin may have a protective effect on bone mass via an antimetabolic mechanism of reduced bone resorption (13, 47). However, how ghrelin balances the relationship between hypertension and lumbar BMD, and whether there are other unknown factors involved in that relationship is not clear, and further mechanisms must be explored.

Our research has two major strengths, a major strength of this study is high-quality, nationally representative data from the NHANES about the United States population. Another strength is that our study may provide new ideas for the control of hypertension in middle-aged and elderly. For hypertension patients, blindly lowering blood pressure does not mean better body health. Keeping blood pressure at a level that maintains body health may be the best choice. Some limitations existed in our study. Firstly, it had a cross-sectional design, and so any argument of causation is weak, which may cause the real causal relationship to not be correctly evaluated. Secondly, there were still some unknown factors, although we adjusted for confounding factors that could affect the results as much as possible. The existence of unknown factors may cause deviations from the current results. Thirdly, we did not include data on total hip bone mineral density, which may have an impact on the results due to the possibility of differences in the association between hypertension and bone density at different bone density measurement locations. Besides, the mechanism underlying the relationship between BMD and hypertension was unclear, which may lead to insufficient evidence for our conclusions. Finally, our subjects were limited to males older than 50 years and postmenopausal females in the United States, which may restrict the ability to extrapolate the conclusions.

## Conclusion

Lumbar BMD was higher in both males and females with hypertension than in those without. Meanwhile, there was a positive association between lumbar BMD and SBP, and a negative association between lumbar BMD and DBP in males and females. Additionally, the proportion of participants with lumbar osteoporosis and low bone mass was slightly but significantly lower in the group of males with hypertension than in the control group.

## Data availability statement

Publicly available datasets were analyzed in this study. This data can be found at: https://www.cdc.gov/nchs/nhanes/index.htm.

## Ethics statement

The studies involving human participants were reviewed and approved by National Center for Health Statistics (NCHS) Research Ethics Review Board. The patients/participants provided their written informed consent to participate in this study.

## Author contributions

SL and JL designed and conducted the study, performed the data analysis, wrote the drafting manuscript, and performed the manuscript review. SL, LL, AF, TH, CC, NH, and LH performed the data acquisition. SL, LL, AF, TH, CC, NH, LH, and JL conducted the data interpretation. JL, SL, LL, AF, and TH revised the manuscript content. All authors approved the final version of the manuscript and approved the decision to submit the manuscript for publication.
