# Supplementary material for: The role of hypertension in bone mineral density among males older than 50 years and postmenopausal females: evidence from the US National Health and Nutrition Examination Survey, 2005–2010
Source: Front Public Health. 2023 Jun 15;11:1142155. doi: 10.3389/fpubh.2023.1142155 (PMC10311208; doi:10.3389/fpubh.2023.1142155)
Supplement: Supplementary file 1 [file Table_1.pdf]

sTable 1 Mean BMD (g/cm^2) according to the type of hypertension by excluding participants with drugs used for hypertension in NHANES 2005 to 2010

	Control	Any hypertension	*p*-value
Femur neck BMD (g/cm^2)			
Male			
Model 1	0.809 ± 0.004	0.820 ± 0.006	0.115
Model 2	0.812 ± 0.004	0.817 ± 0.006	0.549
Model 3	0.812 ± 0.004	0.818 ± 0.006	0.404
Female			
Model 1	0.738 ± 0.005	0.726 ± 0.005	0.115
Model 2	0.731 ± 0.005	0.735 ± 0.006	0.573
Model 3	0.730 ± 0.005	0.736 ± 0.006	0.400
Lumbar spine BMD (g/cm^2)			
Male			
Model 1	1.039 ± 0.005	1.081 ± 0.006	<0.001
Model 2	1.047 ± 0.005	1.071 ± 0.007	0.008
Model 3	1.046 ± 0.005	1.072 ± 0.006	0.002
Female			
Model 1	0.943 ± 0.007	0.953 ± 0.006	0.199
Model 2	0.938 ± 0.006	0.959 ± 0.006	0.016
Model 3	0.937 ± 0.006	0.960 ± 0.006	0.009

Values are the means ± standard error by weighted general linear models.

Model 1: adjusted without any variables.

Model 2: adjusted for age, race/ethnicity, and BMI.

Model 3: adjusted for age, race/ethnicity, marriage, BMI, family PIR, education, recent smoking status, physical activity, total calcium, arthritis, congestive heart failure, coronary heart disease, angina/angina pectoris, heart attack, and stroke.

sTable 2 Multivariate linear regression of association between hypertension and BMD in males and females after excluding participants with drugs used for hypertension

	Male			Female		
	β	95%CI	p	β	95%CI	p
Femur neck BMD g/cm^2						
Model 1	0.011	-0.003, 0.025	0.115	-0.012	-0.027, 0.003	0.115
Model 2	0.005	-0.011, 0.020	0.549	0.004	-0.010, 0.018	0.573
Model 3	0.006	-0.009, 0.002	0.404	0.006	-0.008, 0.020	0.400
Lumbar spine BMD g/cm^2						
Model 1	0.043	0.028, 0.058	<0.001	0.010	-0.006, 0.026	0.199
Model 2	0.024	0.006, 0.041	0.008	0.020	0.004, 0.037	0.016
Model 3	0.026	0.010, 0.042	0.002	0.023	0.006, 0.040	0.009

Model 1: adjusted without any variables.

Model 2: adjusted for age, race/ethnicity, and BMI.

Model 3: adjusted for age, race/ethnicity, marriage, BMI, family PIR, education, recent smoking status, physical activity, total calcium, arthritis, congestive heart failure, coronary heart disease, angina/angina pectoris, heart attack, and stroke.

sTable 3 Mean BMD (g/cm^2) according to the type of hypertension by excluding participants with thyroid problems in NHANES 2005 to 2010

	Control	Any hypertension	p-value
Femur neck BMD (g/cm^2)			
Male			
Model 1	0.810 ± 0.004	0.822 ± 0.005	0.062
Model 2	0.814 ± 0.004	0.818 ± 0.005	0.595
Model 3	0.813 ± 0.004	0.818 ± 0.005	0.470
Female			
Model 1	0.742 ± 0.006	0.732 ± 0.006	0.243
Model 2	0.734 ± 0.005	0.742 ± 0.006	0.288
Model 3	0.733 ± 0.005	0.742 ± 0.006	0.212
Lumbar spine BMD (g/cm^2)			
Male			
Model 1	1.039 ± 0.006	1.081 ± 0.006	<0.001
Model 2	1.049 ± 0.005	1.070 ± 0.006	0.012
Model 3	1.048 ± 0.005	1.072 ± 0.006	0.005
Female			
Model 1	0.941 ± 0.007	0.957 ± 0.007	0.119
Model 2	0.936 ± 0.007	0.963 ± 0.006	0.004
Model 3	0.931 ± 0.007	0.964 ± 0.006	0.003

Values are the means ± standard error by weighted general linear models.

Model 1: adjusted without any variables.

Model 2: adjusted for age, race/ethnicity, and BMI.

Model 3: adjusted for age, race/ethnicity, marriage, BMI, family PIR, education, recent smoking status, physical activity, total calcium, arthritis, congestive heart failure, coronary heart disease, angina/angina pectoris, heart attack, and stroke.

sTable 4 Multivariate linear regression of association between hypertension and BMD in males and females after excluding participants with thyroid problems

	Male			Female		
	β	95%CI	p	β	95%CI	p
Femur neck BMD g/cm^2						
Model 1	0.012	-0.001, 0.025	0.062	-0.010	-0.026, 0.007	0.243
Model 2	0.004	-0.011, 0.019	0.595	0.008	-0.007, 0.022	0.288
Model 3	0.005	-0.010, 0.019	0.470	0.009	-0.006, 0.024	0.212
Lumbar spine BMD g/cm^2						
Model 1	0.042	0.028, 0.056	<0.001	0.016	-0.006, 0.026	0.119
Model 2	0.021	0.005, 0.037	0.012	0.027	0.009, 0.045	0.004
Model 3	0.023	0.008, 0.039	0.005	0.029	0.011, 0.048	0.003

Model 1: adjusted without any variables.

Model 2: adjusted for age, race/ethnicity, and BMI.

Model 3: adjusted for age, race/ethnicity, marriage, BMI, family PIR, education, recent smoking status, physical activity, total calcium, arthritis, congestive heart failure, coronary heart disease, angina/angina pectoris, heart attack, and stroke.

sTable 5 Mean BMD (g/cm^2) according to the type of hypertension by excluding participants with taking prednisone or cortisone daily in NHANES 2005 to 2010

	Control	Any hypertension	*p*-value
Femur neck BMD (g/cm^2)			
Male			
Model 1	0.811 ± 0.003	0.821 ± 0.005	0.117
Model 2	0.815 ± 0.004	0.818 ± 0.005	0.677
Model 3	0.814 ± 0.004	0.818 ± 0.005	0.556
Female			
Model 1	0.742 ± 0.006	0.732 ± 0.006	0.954
Model 2	0.734 ± 0.005	0.742 ± 0.006	0.116
Model 3	0.733 ± 0.005	0.742 ± 0.006	0.110
Lumbar spine BMD (g/cm^2)			
Male			
Model 1	1.039 ± 0.005	1.082 ± 0.005	<0.001
Model 2	1.049 ± 0.005	1.071 ± 0.006	0.006
Model 3	1.048 ± 0.005	1.072 ± 0.006	0.003
Female			
Model 1	0.941 ± 0.007	0.957 ± 0.007	0.016
Model 2	0.936 ± 0.007	0.963 ± 0.006	0.003
Model 3	0.935 ± 0.007	0.964 ± 0.006	0.002

Values are the means ± standard error by weighted general linear models.

Model 1: adjusted without any variables.

Model 2: adjusted for age, race/ethnicity, and BMI.

Model 3: adjusted for age, race/ethnicity, marriage, BMI, family PIR, education, recent smoking status, physical activity, total calcium, arthritis, congestive heart failure, coronary heart disease, angina/angina pectoris, heart attack, and stroke.

sTable 6 Multivariate linear regression of association between hypertension and BMD in males and females after excluding participants taking prednisone or cortisone daily

	Male			Female		
	β	95%CI	p	β	95%CI	p
Femur neck BMD g/cm^2						
Model 1	0.010	-0.003, 0.023	0.117	0	-0.014, 0.013	0.954
Model 2	0.003	-0.011, 0.017	0.677	0.010	-0.003, 0.023	0.116
Model 3	0.004	-0.010, 0.018	0.556	0.011	-0.003, 0.024	0.110
Lumbar spine BMD g/cm^2						
Model 1	0.043	0.030, 0.056	<0.001	0.022	0.004, 0.040	0.016
Model 2	0.022	0.007, 0.037	0.006	0.027	0.010, 0.045	0.003
Model 3	0.023	0.009, 0.038	0.003	0.030	0.011, 0.048	0.002

Model 1: adjusted without any variables.

Model 2: adjusted for age, race/ethnicity, and BMI.

Model 3: adjusted for age, race/ethnicity, marriage, BMI, family PIR, education, recent smoking status, physical activity, total calcium, arthritis, congestive heart failure, coronary heart disease, angina/angina pectoris, heart attack, and stroke.
